# Sequential surgery after conversion therapy for advanced hepatocellular carcinoma: Evidence, controversies, and perspectives

**DOI:** 10.1016/j.iliver.2026.100272

**Published:** 2026-09-28

**Authors:** Yuhao Huo, Hongxiang Jiang, Yuanzhe Liu, Tong Jiang, Cong Ren, Xu Zhang, Boyu Wu, Haowen Tang

**Affiliations:** a Faculty of Hepato-Pancreato-Biliary Surgery, The First Medical Center of Chinese PLA General Hospital, Beijing 100853, China; b Department of Psychology, The Eighth Medical Center of Chinese PLA General Hospital, Beijing 100091, China; c The Second Clinical Medical College, Chongqing Medical University, Chongqing 400010, China; d Clinical School, Chengde Medical College, Chengde 067000, China; e The Norman Bethune-Third Clinical Medical College, Jilin University, Changchun, Jilin 130000, China

**Keywords:** Hepatocellular carcinoma, Conversion therapy, Immune checkpoint inhibitor, Antiangiogenic targeted drug, Sequential surgery, Liver transplantation

## Abstract

Hepatocellular carcinoma (HCC) often has an insidious onset. Therefore, most patients with HCC are diagnosed at intermediate-to-advanced stages, and upfront radical resection cannot be performed. Systemic regimens that combine immune checkpoint inhibitors (ICIs) with antiangiogenic targeted drugs (AATDs) have established conversion therapy followed by sequential surgery as an important research direction for initially unresectable HCC. The aim of conversion therapy is not only to achieve radiographic response and technical resectability but also to reduce tumor viability, identify patients with favorable tumor biology, and gain durable oncologic benefit. Current evidence supports the combination of ICIs and AATDs as the backbone of conversion therapy, with locoregional therapies, including transarterial chemoembolization, hepatic arterial infusion chemotherapy, and stereotactic body radiotherapy, selectively integrated to address heavy intrahepatic tumor burden, control macrovascular thrombus, or treat residual viable lesions. The decision to proceed with sequential surgery should be made after a successful conversion is confirmed by a multidisciplinary evaluation incorporating tumor necrosis, hepatic functional reserve, future liver remnant volume, overall condition, perioperative drug-related risks, and patient preference. This review focuses on identifying the conversion-specific information required to guide surgical decision-making. Additionally, this study aimed to evaluate conversion rate, R0 resection, pathological response, and postoperative outcomes, explain why high objective response rates may not translate into improved postoperative survival, and provide a practical framework integrating tumor biology, hepatic reserve, perioperative risk, and patient preference.

## Abbreviations

AATDs: antiangiogenic targeted drugs
AFP: alpha-fetoprotein
CI: confidence interval
BCLC: barcelona Clinic Liver Cancer
CNLC: china Liver Cancer
HAIC: hepatic arterial infusion chemotherapy
HBV: hepatitis B virus
HCC: hepatocellular carcinoma
HR: hazard ratio
ICI: immune checkpoint inhibitors
IRF: independent review facility
ITT: intention-to-treat
MPR: major pathological response
mRECIST: modified Response Evaluation Criteria in Solid Tumors
NR: not reported
ORR: objective response rate
OS: overall survival
pCR: pathological complete response
PFS: progression-free survival
PIVKA-II: protein induced by vitamin K absence or antagonist-II
PVTT: portal vein tumor thrombosis
RCT: randomized controlled trial
RFS: recurrence-free survival
SBRT: stereotactic body radiotherapy
TACE: transarterial chemoembolization
TACE-PFS: the trial-defined TACE-specific progression-free survival endpoint
TKI: tyrosine kinase inhibitor
VEGF: vascular endothelial growth factor

## 1 Introduction

Primary hepatic malignancies impose a substantial global health burden with high incidence and mortality rates. In China, recent epidemiological estimates indicate an annual incidence of 367,700 new diagnoses and 316,500 cancer-specific deaths.^1^ Hepatocellular carcinoma (HCC) is the predominant pathological type of primary liver cancer, accounting for 75.0%–90.0% of all cases.^2^^,^^3^ HCC oncogenesis is closely associated with underlying factors, including chronic hepatitis B or C virus infection, alcohol abuse, metabolic dysfunction-associated steatotic liver disease, aflatoxin ingestion, and cirrhotic remodeling, contributing to variations in disease distribution across different geographical regions and etiologies. 4, 5, 6 Because early-stage lesions typically lack specific clinical symptoms, most patients present with intermediate-to-advanced disease, which rules out initial radical treatments.^7^ According to the Chinese Expert Consensus on Multidisciplinary Treatment of Liver Cancer (2025 edition) and the updated Barcelona Clinic Liver Cancer (BCLC) staging system in 2026, advanced HCC is defined as HCC with radiologically evident vascular tumor thrombus or extrahepatic metastasis, corresponding to China Liver Cancer Staging (CNLC) stage IIIa/IIIb or BCLC stage C disease.^8^^,^^9^

Upon initial presentation, only <30% of patients with HCC are candidates for curative-intent surgical options, leaving those with advanced stages with limited radical alternatives and historically dismal outcomes.^10^ The standard recommendations for CNLC stage IIIa disease, particularly when complicated by portal vein tumor thrombosis (PVTT) involving the main trunk, focus on transarterial chemoembolization (TACE) or TACE combined with systemic therapies, whereas primary surgical resection is restricted to highly selected scenarios following selected multidisciplinary reviews. A combination of systemic agents and localized palliative interventions is a preferred option for patients with CNLC stage IIIb characterized by systemic metastases.^3^ The European Association for the Study of the Liver guidelines and the BCLC staging system recommend systemic therapy for unresectable or BCLC stage C disease, with treatment selection made based on tumor burden, hepatic function, performance status, and patient goals. 9, 10, 11, 12 With advances in systemic therapy, immune checkpoint inhibitors (ICIs) combined with antiangiogenic targeted drugs (AATDs) have become important first-line options for unresectable HCC in several guidelines. ^3^^,^^11^, ^12^, ^13^ This therapeutic paradigm shift has improved overall survival (OS) and opened up new possibilities for downstaging, local radical management, and sequential surgical resection in selected patients with advanced HCC.^14^

Building on the established 2024 framework for sequential surgery after conversion therapy based on the combination of ICIs and AATDs,^15^^,^^16^ this review aimed to synthesize recent phase III and conversion-specific evidence, including EMERALD-1, LEAP-012, TALENTACE, SILENSES, and updated IMbrave150 and IMbrave050 results, and evaluate conversion rate, R0 resection, pathological response, and postoperative recurrence-free survival (RFS)/OS. Furthermore, this review aimed to develop a practical decision framework that integrates residual tumor viability, tumor biology, hepatic functional reserve, perioperative risk, and patient preference, with particular attention given to the distinction between anatomical resectability and durable oncologic benefit, interpretation of conversion-specific endpoints across heterogeneous studies, and clinical implications of recent randomized data for postoperative treatment. This review may provide a focused, decision-oriented synthesis of current strategies for conversion and sequential surgery in advanced HCC.

## 2 Concept and theoretical basis of conversion therapy

### 2.1 Definition and biological rationale of conversion therapy

Conversion therapy is the use of systemic or locoregional treatment to convert initially unresectable HCC into a resectable state, providing patients with the opportunity to undergo curative-intent surgery. The core meaning of conversion therapy includes two dimensions. The first is conversion to surgical resectability, as antitumor treatment reduces tumor volume and promotes vascular tumor thrombus regression, allowing an adequate functional liver remnant (≥35% and ≥45% of standard liver volume in noncirrhotic and cirrhotic patients, respectively) and meeting surgical criteria. The second is conversion to oncologic benefit, as treatment induces tumor necrosis, reduces viable tumor activity, and improves the tumor immune microenvironment, thereby reducing postoperative recurrence and improving long-term survival. 15, 16, 17 Therefore, conversion therapy should extend beyond simple tumor downsizing followed by resection to include selecting patients with relatively favorable tumor biology who can tolerate curative-intent therapy and may derive a durable survival benefit based on treatment response.

The rationale for using the combination of ICIs and AATDs as the backbone of conversion therapy stems from the interaction between the HCC tumor microenvironment and angiogenic pathways. HCC is typically hypervascular. Proangiogenic factors, such as vascular endothelial growth factor (VEGF), promote abnormal angiogenesis and tumor growth while contributing to an immunosuppressive microenvironment by inhibiting dendritic cell maturation, recruiting regulatory T cells and myeloid-derived suppressor cells, and limiting effector T-cell infiltration. 18, 19, 20, 21 Anti-VEGF antibodies can promote vascular normalization, facilitate immune-cell entry into tumor tissue, and alleviate local immunosuppression. Furthermore, inhibitors targeting programmed cell death protein 1, programmed death-ligand 1, or cytotoxic T-lymphocyte-associated antigen 4 can release T-cell exhaustion and restore antitumor immunity. The combination of these two agent classes may exert synergistic effects on tumor shrinkage, arterial enhancement reduction, tumor thrombus regression, and potential micrometastasis control. 18, 19, 20^,^^22^, ^23^, ^24^, ^25^

The combination of locoregional therapy and systemic treatment has a sound mechanistic basis. TACE can increase tumor necrosis by blocking blood supply to the tumor and delivering local chemotherapy. Hepatic arterial infusion chemotherapy (HAIC) can increase local drug concentrations in patients with high intrahepatic tumor burden and PVTT. Furthermore, stereotactic body radiotherapy (SBRT) can accurately irradiate a vascular tumor thrombus or residual viable lesions. These locoregional approaches may release tumor-associated antigens and promote antigen presentation while inducing tumor cell death, thereby providing additional targets for immunotherapy. 26, 27, 28, 29, 30, 31, 32, 33, 34 Conversely, systemic combined immunotherapy and targeted therapy can target micrometastases and extrahepatic lesions that are not covered by local therapy.^17^^,^^21^ However, earlier, more frequent, or more intensive application of locoregional therapy to escalate treatment should be avoided. Excessive TACE or radiotherapy may aggravate liver injury, biliary injury, or portal hypertension, thereby compromising subsequent surgical safety. Therefore, locoregional treatment should be regarded as an intensification and supplement to systemic therapy rather than an indiscriminate add-on.

### 2.2 Clinical assessment and management of conversion therapy

The assessment of conversion therapy should not rely solely on changes in tumor diameter according to the Response Evaluation Criteria in Solid Tumors version 1.1 (RECIST 1.1). HCC may show a marked reduction in arterial enhancement after the combination of ICIs, AATDs, and locoregional therapy, despite the limited change in tumor size. The modified RECIST (mRECIST) more accurately reflects changes in the viable tumor burden.^35^ The dynamic decline in alpha-fetoprotein (AFP) and protein induced by vitamin K absence or antagonist-II (PIVKA-II), decreased PVTT enhancement or regression, extrahepatic disease stability or disappearance, and new lesion absence during therapy are additional indicators of favorable conversion. Simultaneous assessment of Child–Pugh grade, albumin-bilirubin score, indocyanine green retention rate at 15 min (ICG R15), platelet count, coagulation status, portal hypertension, future liver remnant volume, and performance status should be considered for patients being considered for sequential surgery. Conversion therapy can be considered meaningful only when the radiographic findings, tumor markers, and hepatic functional reserve are concordant. ^11^^,^^12^^,^^15^, ^16^, ^17^

Conversion therapy is not equivalent to neoadjuvant therapy. Neoadjuvant therapy is administered before surgery to patients who are technically resectable but have an intermediate or high risk of recurrence. Its purpose is to eradicate micrometastases, reduce the risk of subsequent relapse or metastasis, and improve survival. Conversely, conversion therapy is intended for patients who are initially unresectable or unsuitable for radical treatment. Its main purpose is to restore the possibility of curative surgery. The two strategies differ in target populations, treatment goals, treatment durations, and surgical timing criteria. Sequential surgery after conversion therapy for advanced HCC is a dynamic selection strategy. Patients who respond to conversion therapy enter a curative-intent pathway, whereas those who do not respond or progress should promptly switch to alternative systemic or locoregional control strategies to avoid delays due to indiscriminate watchful waiting. 14, 15, 16, 17

From the perspective of the disease course, conversion therapy for advanced HCC is characterized by parallel tumor treatment and liver disease management. HCC usually develops in cases of chronic hepatitis or cirrhosis. The liver is the tumor-harboring organ, and its function determines whether treatment and surgery can be tolerated. Although the combination of ICIs and AATDs can achieve relatively high response rates, immune-mediated hepatitis, post-TACE hepatic function fluctuation, cholestasis, and worsening portal hypertension may cause patients approaching resectability to lose the surgical opportunity. Therefore, antiviral therapy, liver-protective treatment, nutritional support, and portal hypertension and gastroesophageal varices evaluation should be performed throughout conversion therapy. Particularly, the risk of gastrointestinal bleeding should be thoroughly evaluated and managed as needed before the use of bevacizumab or other anti-VEGF agents. In patients with hepatitis B virus (HBV)-related HCC, HBV DNA replication should be continuously controlled before and after systemic therapy to reduce the risk of hepatitis reactivation and treatment interruption.^11^^,^^12^^,^^36^

From an oncological perspective, conversion therapy functions as a biological selection. Advanced HCC is highly heterogeneous, and patients receiving the same combination of ICIs and AATDs may experience rapid response, stable disease, mixed response, pseudoprogression, and primary resistance. Early reduction in AFP or PIVKA-II, regression of tumor thrombus, and stability of extrahepatic lesions usually indicate treatment sensitivity. Conversely, the presence of new lesions, persistent elevation of tumor markers, and progression of nontarget lesions during therapy indicate a more aggressive biology. The long-term benefit may be limited in such patients even if the local lesion becomes technically resectable.^15^^,^^16^^,^^37^^,^^38^ Therefore, successful conversion therapy should be determined based on dynamic concordance among imaging, serological markers, and clinical status, rather than based on a single radiographic partial response or local shrinkage alone.

## 3 Conversion treatment regimens and evidence advances

The selection of a conversion regimen is a dynamic multidisciplinary process that considers several factors, including tumor biology, hepatic functional status, overall condition, treatment goals, and socioeconomic factors. Because of their relatively high response rates, ICI-based combination regimens have accumulated substantial evidence in unresectable or advanced HCC and have become important strategies in conversion therapy research.

### 3.1 Mainstream regimens and evidence base

The combination of ICIs and AATDs has shown considerable antitumor activity in multiple clinical studies. Their potential advantages include tumor shrinkage, arterial enhancement reduction, vascular tumor thrombus regression, and increased subsequent curative treatment opportunities. Table 1 summarizes the representative efficacy data for commonly used combined ICIs, AATDs, and locoregional regimens.

**Table 1:** Representative efficacy data for ICI-, antiangiogenic-, and locoregional-based regimens in unresectable or intermediate-to-advanced HCC.

Treatment regimen (reference)	Median OS	Median PFS/TACE-PFS	OS rate	RFS/PFS rate	ORR	Study design and population	Sample size (experimental vs. control)
Atezolizumab + bevacizumab vs. sorafenib^23^^,^^39^	19.2 vs. 13.4 months	6.9 vs. 4.3 months	1-year: 67% vs. 56%; 18 months: 52% vs. 40%	6 months PFS: 54.5% (experimental arm)	33.2% vs. 13.3% (IRF; mRECIST)	Phase III RCT; first-line unresectable HCC	501 (336 vs. 165)
TKI + anti-PD-1 antibody combinations^40^	NR	NR	1-year: 90% (operated, *n* = 10)	1-year RFS: 80.0% (operated, *n* = 10)	46% (full cohort)	Prospective consecutive cohort; initially unresectable HCC	63 total; 10 underwent R0 resection
Sintilimab + bevacizumab biosimilar (IBI305) vs. sorafenib (ORIENT-32)^41^	NR vs. 10.4 months in the first interim analysis	4.6 vs. 2.8 months	NR (OS data immature at first interim analysis)	NR	21%	Phase II/III RCT; first-line HBV-related unresectable HCC	571 (380 vs. 191)
Camrelizumab + apatinib (RESCUE, first-line cohort)^42^	NR in the primary publication	5.7 months	12 months: 74.7%	NR	34.3% (IRF; RECIST 1.1)	Nonrandomized, open-label phase II trial; first-line advanced HCC	70 (first-line cohort; total trial *n* = 190, including 120 second-line patients)
Nivolumab 1 mg/kg + ipilimumab 3 mg/kg (arm A)^43^	22.2 months	NR	3-year: 42%; 5 years: 29%	NR	34% (investigator-assessed, RECIST 1.1)	Randomized phase I/II expansion cohort; previously sorafenib-treated advanced HCC	50 in arm A (total trial *n* = 148 across 3 arms)
Durvalumab + bevacizumab + TACE vs. TACE^44^^,^^45^	29.9 vs. 33.3 months; HR, 1.10 (95% CI: 0.87–1.39); no OS benefit	15.0 vs. 8.2 months; HR, 0.77 (95% CI: 0.61–0.98)	NR	NR	43.6% vs. 29.6% (RECIST 1.1)	Phase III RCT; unresectable, embolization-eligible HCC	616 (204 triplet vs. 207 durvalumab vs. 205 placebo; triplet vs. placebo comparison)
Pembrolizumab + lenvatinib + TACE vs. dual placebo + TACE (LEAP-012)^46^	NR; HR, 0.80 (95% CI: 0.57–1.11); *p* = 0.087, not significant	14.6 vs. 10.0 months; HR, 0.66 (95% CI: 0.51–0.84)	24 months: 75% vs. 69%	NR	71.3% (mRECIST; experimental arm)	Phase III RCT; unresectable, nonmetastatic HCC	480 (237 vs. 243)
Atezolizumab + bevacizumab + on-demand TACE vs. on-demand TACE (TALENTACE)^47^	34.53 vs. 35.38 months (immature); HR, 0.96 (99.62% CI: 0.58–1.58); no demonstrated OS benefit	TACE-PFS 11.30 vs. 7.03 months; HR, 0.71 (95% CI: 0.55–0.92)	NR	NR	NR in the published report	Open-label phase III RCT; systemically untreated, intermediate-to-high-burden unresectable HCC	342 (171 vs. 171)

Abbreviations: CI, confidence interval; HCC, hepatocellular carcinoma; HR, hazard ratio; ICI, immune checkpoint inhibitor; IRF, independent review facility; mRECIST, modified Response Evaluation Criteria in Solid Tumors; NR, not reported or not reached; ORR, objective response rate; OS, overall survival; PFS, progression-free survival; RCT, randomized controlled trial; RFS, recurrence-free survival; TACE, transarterial chemoembolization; TACE-PFS, the trial-defined TACE-specific progression-free survival endpoint; TKI, tyrosine kinase inhibitor.

### 3.2 Locoregional combination strategies

In patients with advanced HCC, the addition of locoregional therapy to the combination of ICIs and AATDs may improve local control, increase tumor necrosis, and shorten the time to resectability.^26^^,^^27^ TACE, HAIC, and SBRT can serve as intensification strategies based on systemic therapy, particularly for patients with high intrahepatic tumor burden, clear need for local control of tumor thrombus, or concentrated residual viable lesions after systemic treatment.

TACE-based combinations have been tested in several phase III trials. EMERALD-1 demonstrated that TACE plus durvalumab and bevacizumab significantly prolonged PFS and time to progression compared with the TACE-based control, with an objective response rate (ORR) of 43.6% in the triplet arm.^44^ Furthermore, LEAP-012 demonstrated a PFS advantage for pembrolizumab plus lenvatinib combined with TACE and reported an ORR of 71.3% in the experimental arm.^46^ TALENTACE demonstrated that atezolizumab plus bevacizumab combined with on-demand TACE significantly prolonged investigator-assessed TACE-PFS compared with on-demand TACE alone (median, 11.30 vs. 7.03 months; HR, 0.71; 95% CI: 0.55–0.92; *p* = 0.0089). The investigator-assessed PFS per RECIST 1.1 was 10.32 vs. 6.37 months (HR, 0.64; 95% CI: 0.50–0.82). The OS remained immature at the first interim analysis (median, 34.53 vs. 35.38 months; HR, 0.96; 99.62% CI: 0.58–1.58), with no demonstrated OS benefit.^47^ These trials enrolled different populations, including those with embolization-eligible, unresectable HCC with limited portal vein invasion, those with nonmetastatic HCC without macrovascular invasion or extrahepatic spread, and those with systemically untreated intermediate-to-high tumor burden, unresectable HCC. Although not identical to the results of conversion-to-surgery studies in advanced HCC, they provide evidence that systemic therapy combined with locoregional treatment can improve disease control. Other locoregional modalities may be combined with immunotherapy and targeted therapy. HAIC and SBRT can be used to intensify the treatment of the primary lesion, PVTT, or residual viable lesions, thereby potentially improving local control and the conversion success rate.^17^^,^^30^^,^^33^^,^^34^

The optimal sequence and timing of ICIs, AATDs, and locoregional therapy in advanced HCC remain undefined. Current evidence and clinical experience support the use of a stepwise combination strategy. Immunotherapy-targeted therapy can serve as the backbone, and TACE, HAIC, radiotherapy, or other locoregional treatments can be selectively added when treatment response is slow, residual viable tumor is prominent, focal resistance emerges, or rapid tumor thrombus or primary lesion control is required. This on-demand intensification strategy balances sensitivity to systemic therapy and the efficacy of local treatment while improving tolerability when multiple antitumor modalities are combined.

### 3.3 Adverse events and safety management

Although these regimens are effective, clinical decision-making must balance conversion efficiency and safety. Findings from various ICI-AATD-based or multimodal studies are inconsistent. LEAP-002 did not meet the prespecified statistical significance threshold for its primary survival endpoints, and COSMIC-312 demonstrated improved PFS but did not demonstrate a significant OS advantage. Both were associated with relatively high rates of grade ≥3 adverse events. CARES-310 and APOLLO were positive studies. However, adverse event management remains a key issue in clinical applications. 48, 49, 50, 51

Immune-related adverse events caused by immune activation against normal tissues are the most characteristic toxicities. These reactions can involve almost any organ system and are common in the skin (rash or pruritus), gastrointestinal tract (diarrhea or colitis), and endocrine organs (thyroid dysfunction or hypophysitis). Although severe pulmonary (pneumonitis) and cardiac (myocarditis) toxicities are relatively uncommon, high vigilance is required due to their potentially serious consequences.

Adverse events should be graded according to the Common Terminology Criteria for Adverse Events version 5.0. Safety management during the peritreatment period should be stratified according to the toxicity source. For immune-related hepatitis, pneumonitis, colitis, and endocrine dysfunction, whether ICIs are withheld and whether glucocorticoids or other immunosuppressive therapies should be promptly initiated for moderate-to-severe events should be guided by the Common Terminology Criteria for Adverse Events grade. For anti-VEGF-related hypertension, proteinuria, bleeding, thrombosis, hand-foot syndrome, and impaired wound healing, the main measures are dose modification, temporary discontinuation, and symptomatic treatment. For hepatic dysfunction, biliary injury, myelosuppression, and radiation-related reactions after TACE, HAIC, or SBRT, surgery should be considered only after the acute injury has resolved and the hepatic reserve has been reassessed.^15^

## 4 Concept, evaluation criteria, and clinical evidence for sequential surgery following conversion therapy

### 4.1 Definition and evaluation criteria

Sequential surgery following conversion therapy is a natural extension of the conversion therapy framework, which refers to curative-intent surgery, including hepatectomy or liver transplantation, after conversion of initially unresectable HCC to a resectable or transplantable state through systemic or locoregional therapy and after multidisciplinary evaluation. The aim of sequential surgery is to translate the disease control achieved during conversion therapy into long-term survival benefit. Appropriate surgical conditions include adequate future liver remnant volume after tumor resection (≥35% and ≥45% of standard liver volume in noncirrhotic and cirrhotic patients, respectively), Child–Pugh class A or B, ICG R15 < 20%, preserved hepatic inflow and outflow structures with unobstructed blood flow, intact biliary anatomy and bile drainage, Eastern Cooperative Oncology Group performance status 0–1, and American Society of Anesthesiologists class ≤ III.^17^

The core issues in sequential surgery are determining whether curative surgery can provide meaningful benefit and identifying an appropriate intervention window. Surgical decision-making should avoid premature surgery after insufficient conversion and excessive waiting for a deeper response that may cause loss of the resection window. Clinical assessment should integrate treatment response, tumor biology, hepatic reserve, future liver remnant volume, surgical complexity, drug-related risks, and overall condition. Sequential surgery aims to eradicate residual viable tumors and potentially resistant clones through curative resection while providing pathological information for response assessment, recurrence risk stratification, and postoperative adjuvant treatment decisions.

### 4.2 Clinical evidence for sequential surgery

Current evidence indicates that sequential surgery after conversion therapy based on the combination of ICIs and AATDs confers a survival benefit in selected patients with advanced HCC. Potential mechanisms underlying this survival benefit include systemic therapy-induced tumor shrinkage, necrosis, and immune microenvironment remodeling, followed by surgical eradication of local residual lesions and resistant clones, with pathological response clarifying treatment sensitivity. The phase III HIMALAYA trial evaluated the STRIDE regimen, consisting of one priming dose of tremelimumab, an anti-cytotoxic T-lymphocyte-associated antigen 4 antibody, followed by scheduled maintenance with durvalumab, an anti-programmed death-ligand 1 antibody. The regimen was associated with improved survival and delayed deterioration of quality of life among patients with unresectable HCC.^52^ Although the phase III HIMALAYA trial was not designed as a conversion surgery trial, it supports the role of immunotherapy in advanced HCC and provides a rationale for further studies on conversion and sequential treatment.

Studies on HCC with PVTT have compared sequential surgery after conversion therapy based on combined immunotherapy and targeted therapy with surgery alone. A previous study of 100 patients, which included 36 in the conversion surgery group and 64 in the upfront surgery group, showed that >90% of patients in the conversion surgery group had Vp3–4 portal vein invasion. The 6-, 12-, and 24-month RFS rates were significantly higher in the conversion surgery group than in the upfront surgery group (80.3% vs. 36.5%, 65.4% vs. 29.4%, and 56.0% vs. 21.0%, respectively; *p* < 0.05).^53^ A separate prospective single-center study of 100 patients with initially unresectable HCC who underwent radical surgery after successful conversion with a programmed cell death protein 1 antibody plus a targeted agent showed that 24.0% of patients achieved postoperative pathological complete response (pCR), the median RFS was 32.4 months, and the 1-, 3-, and 5-year OS rates were 98.0%, 83.1%, and 74.5%, respectively.^37^ These outcomes should not be interpreted as a conversion success rate and are susceptible to selection bias because this cohort was defined by successful conversion followed by surgery.

Other studies have reported similar trends. A phase II trial conducted by the hepatobiliary team at the First Affiliated Hospital of Nanjing Medical University evaluated the efficacy and safety of perioperative camrelizumab plus apatinib in patients with HCC. A total of 18 patients with CNLC stage IIb/IIIa HCC received three cycles of ICIs combined with AATDs before surgery and eight cycles of postoperative adjuvant therapy. The ORRs according to the RECIST 1.1 and mRECIST were 16.7% and 33.3%, respectively. The major pathological response (MPR) and pCR rates among the operated patients were 17.6% and 5.9%, respectively. The 1-year RFS rate was 53.8%, and safety was manageable.^54^ A retrospective study of 613 patients with initially unresectable HCC from Peking Union Medical College Hospital showed that 135 met the conversion surgery criteria during ICI-based combination therapy, and 128 were included in the final analysis (74 surgical vs. 54 nonsurgical therapy). The median PFS was 29.4 months, and the 3-year OS rate was 79.0% in the surgical group, whereas the median PFS and OS were 11.2 and 25.4 months, respectively, in the nonsurgical group. These findings indicate that surgery after conversion benefits carefully selected patients. However, prospective randomized evidence is required.^38^

Recent studies have directly addressed conversion and sequential surgery. In the SILENSES phase II expansion trial, sintilimab plus lenvatinib was used as conversion therapy in 120 patients with advanced unresectable HCC. Among them, 67 patients (56%) achieved a successful conversion, and 60 subsequently underwent surgical resection. The median OS of the overall cohort after a median follow-up of 41.0 months was 36.0 months, with a 5-year survival rate of 42.6%. Among resected patients, the median RFS was 40.0 months, and the 5-year survival rate was 73.9%.^55^ A prospective study of 76 patients with HCC and PVTT who underwent surgery after conversion therapy with programmed cell death protein 1 inhibitors plus tyrosine kinase inhibitors showed a median RFS of 21 months and a median OS of 62 months, with 3-year RFS and OS rates of 42.5% and 72.4%, respectively. In this study, no patient died from conversion therapy or surgery.^56^ These findings are consistent with earlier retrospective and single-arm evidence, although neither study was randomized and both were conducted in China. Table 2 presents the conversion-specific outcomes of the representative studies.

**Table 2:** Conversion-specific outcomes in representative studies of conversion therapy followed by sequential surgery.

Study (reference)	Design and population	Conversion success rate	Surgical resection rate	R0 resection rate	pCR rate	MPR rate	Postoperative RFS	Postoperative OS
Zhang et al., 2023^25^	Single-arm phase II trial; unresectable BCLC-B/C HCC (*n* = 56)	55.4% (31/56)	37.5% (21/56)	85.7% (18/21 operated); ITT: 32.1% (18/56)	38.1% (8/21 operated); ITT: 14.3% (8/56)	NR	12 months RFS 47.6%; median RFS 11.6 months (operated)	Median OS 23.9 months (full cohort); median postoperative OS 31.4 months (operated)
Zhu et al., 2021^40^	Prospective consecutive cohort; initially unresectable HCC (*n* = 63)	NR	15.9% (10/63)	100% (10/10 operated)	60% (6/10 operated)	NR	12 months RFS 80.0% (operated); 8/9 patients recurrence-free at a median follow-up of 11.2 months	12 months OS 90.0% after therapy initiation (operated); full cohort OS NR
SILENSES, 2026^55^	Single-arm phase II expansion trial; advanced unresectable HCC (*n* = 120)	56% (67/120)	50.0% (60/120)	97% (58/60 operated)	35% (21/60 operated)	NR	Median RFS 40 months (operated)	Median OS 36.0 months (full cohort); 5-year OS 42.6% (full cohort); 5-year survival 73.9% (operated); median follow-up 41.0 months
Gao et al., 2026^56^	Prospective single-center study; HCC with PVTT (*n* = 76, surgical cohort)	NR (all analyzed after surgery)	NR (surgical cohort by design)	NR	NR	NR	Median RFS 21 months; 3-year RFS 42.5%	Median OS 62 months; 3-year OS 72.4%; no deaths attributable to conversion therapy or surgery
PUMCH cohort, 2025^38^	Retrospective cohort; initially unresectable HCC (*n* = 613 screened; 135 met conversion surgery criteria; 128 were included in the final analysis, 74 surgery vs. 54 nonsurgery)	22.0% (135/613)	54.8% (74/135 conversion-eligible); 12.1% (74/613) overall	91.9% (68/74 operated)	31.1% (23/74 operated)	NR	Median PFS 29.4 vs. 11.2 months (surgery vs. nonsurgery); median RFS after surgery 15.8 months	Median OS NR (surgery) vs. 25.4 months (nonsurgery); 3-year OS 79.0% (surgery)
Cao et al., 2023^53^	Retrospective propensity-score analysis; HCC with PVTT (*n* = 100; 36 conversion surgeries vs. 64 upfront surgery; >90% Vp3–4 in the conversion group)	NR (both groups underwent surgery)	Not applicable (comparison of surgical strategies)	NR	22% (8/36 operated)	38% (14/36 operated)	6/12/24-month RFS: 80.3/65.4/56.0% (conversion) vs. 36.5/29.4/21.0% (upfront surgery), *p* < 0.05	2-year OS: 73.3% (conversion surgery) vs. 38.2% (upfront surgery)
Xia et al., 2022^54^	Single-arm phase II perioperative (neoadjuvant) trial; resectable CNLC IIb/IIIa HCC (*n* = 18)	Not applicable (resectable at baseline)	17/18 underwent surgery	NR	5.9% (1/17 operated)	17.6% (3/17 operated)	1-year RFS 53.8%	NR

Abbreviations: NR, not reported in the cited source; ITT, intention-to-treat; R0, complete macroscopic and microscopic resection; pCR, pathological complete response; MPR, major pathological response; RFS, recurrence-free survival; OS, overall survival; PVTT, portal vein tumor thrombosis; CNLC, China Liver Cancer Staging; BCLC, Barcelona Clinic Liver Cancer. Conversion success rate is defined as the proportion of initially unresectable patients meeting predefined conversion or resectability criteria; surgical resection rate is the proportion who actually underwent resection (denominators in parentheses). Unless otherwise indicated, R0, pCR, and MPR are calculated among operated patients, and RFS/OS refers to the surgical cohort.

### 4.3 Liver transplantation after conversion therapy

Liver transplantation serves as a potential curative strategy within sequential surgery after conversion therapy. Liver transplantation may be considered a curative option for strictly selected patients who meet transplant or downstaging criteria after conversion therapy. Unlike hepatectomy, transplantation can simultaneously treat the tumor and underlying liver disease, particularly cirrhosis. Additionally, it may be more advantageous in patients who cannot tolerate extensive hepatectomy due to limited hepatic reserve, significant portal hypertension, and severe multinodular cirrhotic background. The classic Milan criteria and extended criteria are important foundations for transplant candidate selection and prognostication. Entry into a transplantation pathway after conversion should be determined based on tumor burden, AFP level, macrovascular invasion or extrahepatic metastasis, and stability during the waiting period.^57^^,^^58^

Bridge to transplantation after conversion requires careful evaluation of several issues. First, downstaging depth and durability are important. Radiographic complete or partial response lasting for a period of time, usually 3–6 months, without progression indicates more favorable tumor biology and a relatively controllable risk of posttransplant recurrence. This may represent an appropriate window for transplant candidacy. Previous downstaging studies and the MERITS-LT consortium have shown that patients who meet the United Network for Organ Sharing downstaging criteria or who are strictly selected and successfully downstaged to within transplant criteria can achieve favorable posttransplant outcomes, whereas patients with a very high initial tumor burden, persistent AFP elevation, or Child–Pugh B/C cirrhosis have higher risks of waitlist dropout and posttransplant recurrence. 59, 60, 61 Second, systemic therapy maintenance should be considered during the waiting period based on tumor control, waiting time, and tolerability to prevent tumor progression and waitlist dropout. Third, coordinating peritransplant immunosuppression is a major clinical challenge. ICIs have long half-lives and may increase the risk of rejection or immune-related toxicity after transplantation. Individual patient data meta-analyses and global cohort studies have indicated that a longer ICI washout period is associated with a lower risk of posttransplant rejection. However, no unified discontinuation interval has been established. Based on available evidence, a washout period of at least 4–6 weeks is commonly recommended before transplantation, with longer intervals for agents such as ipilimumab with several half-lives.^62^^,^^63^ An individual patient data meta-analysis of 91 ICI-exposed recipients revealed that allograft rejection occurred in 24 patients (26.4%), and a longer washout period was associated with a lower risk of rejection. A washout period of approximately 3 months may reduce this risk to that of ICI-naive recipients.^62^ A global cohort study of 386 patients showed that graft rejection occurred in 17.5% of patients who received ICIs before transplantation, and a washout of >30 days or 1.5 half-lives was associated with a significantly lower risk of rejection.^63^ These findings indicate that the rejection risk is not negligible and should be balanced against the risk of tumor progression during the waiting period. Furthermore, the drug type, time since the last dose, rejection risk, and tumor progression risk should be considered.^62^^,^^63^ Given the limited experience, such patients should be managed in centers with substantial expertise in liver transplantation and immunotherapy, with decisions made by a multidisciplinary team.

The choice between hepatectomy and liver transplantation should be individualized for patients who achieve a deep response after conversion. If the hepatic function is good, the future liver remnant is adequate, and an R0 resection is expected, hepatectomy is less invasive and avoids lifelong immunosuppression. If the hepatic reserve is marginal (Child–Pugh class B or elevated ICG R15), the tumor is downstaged, but the liver remains affected by multinodular cirrhosis, or significant portal hypertension is present preoperatively, liver transplantation may provide better long-term disease-free survival and quality of life. The washout interval, rejection risk, tumor progression risk during waiting, and donor availability should be incorporated into decision-making for patients previously exposed to ICIs. The choice between these two pathways should be based on multidisciplinary assessment and patient preference. 57, 58, 59, 60, 61, 62, 63

The evidence summarized above has several limitations. Most studies on conversion surgery are single-arm phase II trials, retrospective cohort studies, or small single-center studies without randomized comparators. Additionally, patients who complete conversion therapy and proceed to surgery are inherently selected based on favorable tumor biology, adequate hepatic function, and good performance status. Therefore, the improved outcomes associated with sequential surgery may partly reflect selection bias, lead-time bias, or selective survival bias rather than a direct benefit of resection. Furthermore, the definitions of unresectability, conversion success, and surgical timing, as well as the response criteria (RECIST 1.1 vs. mRECIST) vary across studies. Therefore, prospective randomized trials comparing sequential surgery with continued systemic or locoregional therapy in patients who achieve conversion are needed. Until such evidence is available, surgical decisions should be considered individualized multidisciplinary judgment rather than a proven standard of care.

## 5 Formulation and selection of conversion strategies

### 5.1 General principles for selecting a conversion regimen

The selection of a conversion strategy should move beyond simply pursuing a higher response rate toward determining whether resectability and meaningful benefit can be achieved. Existing studies have shown that ORR, PFS, and conversion resection rates are influenced by enrollment stage, tumor burden, tumor thrombus type, treatment line, response criteria, and follow-up duration. Therefore, clinical priority should not be determined solely by the ORR reported in a single study. The treatment goals for patients with advanced HCC should be clarified first. If the main goal is rapid control of a large intrahepatic lesion or tumor thrombus, synergy between local control and systemic therapy should be emphasized. If the goal is to control extrahepatic metastasis or micrometastasis, the continuity of systemic therapy should be prioritized. If hepatic function is marginal, excessive locoregional therapy, which may damage hepatic reserve, should be avoided. 4, 5, 6^,^^11^^,^^12^^,^^21^

### 5.2 Evaluation of surgical benefit after successful conversion therapy

Successful conversion to technical resectability does not mean that all patients should proceed to surgery immediately. The value of sequential surgery is to translate disease control achieved by drug and locoregional therapies into potential long-term survival rather than to mechanically respond to radiographic improvement. Patients who meet the criteria of hepatic function, future liver remnant, performance status, and tumor control should actively undergo multidisciplinary evaluation for hepatectomy, liver transplantation, or other curative-intent treatments. However, continued systemic therapy or locoregional consolidation may be preferable for patients with progressive extrahepatic metastases, irreversible hepatic dysfunction, need for sacrifice of critical vascular or biliary structures, or excessive comorbidity-related surgical risk. ^11^^,^^12^^,^^15^, ^16^, ^17^

### 5.3 Current controversies and evidence gaps

The inconsistency across ICI-AATD trials reflects differences in study populations and treatment design rather than a simple difference in regimen activity. These trials enrolled patients with different BCLC/CNLC stages, tumor burdens, PVTT extents, extrahepatic disease, hepatic function, and regional HBV prevalence. Additionally, they used different antiangiogenic agents, treatment sequences, comparators, and endpoints. The IMbrave150, CARES-310, and APOLLO trials met their primary survival endpoints, whereas the LEAP-002 and COSMIC-312 trials did not. ^23^^,^^48^, ^49^, ^50^, ^51^^,^^64^ In the EMERALD-1 trial, the addition of bevacizumab to durvalumab plus TACE was associated with improved PFS, although updated follow-up did not demonstrate an OS benefit.^44^^,^^45^ Additionally, the LEAP-012 trial showed a PFS benefit with lenvatinib plus pembrolizumab plus TACE, but its interim OS result was not statistically significant.^46^ These findings support selective locoregional intensification. However, they indicate that efficacy cannot be transferred directly from one regimen, population, or endpoint to another.

High ORRs should not be equated with successful conversion. Conversion surgery requires durable disease control, adequate future liver remnant, preserved vascular and biliary anatomy compatible with R0 resection, adequate hepatic reserve, and favorable tumor biology. 15, 16, 17 A radiographic response may be transient, anatomically unsuitable for curative resection, or associated with toxicity that delays surgery or worsens hepatic function. Nonrandomized comparisons of conversion surgery are confounded by selecting patients with favorable tumor biology and differences in subsequent therapy.^37^^,^^38^^,^^53^ Therefore, the conversion rate, R0 resection, pCR/MPR, postoperative RFS, and OS should be reported and interpreted separately rather than inferred from the ORR.

These distinctions highlight the gaps in the principal evidence. Current studies differ in their definitions of unresectability and successful conversion, often combine Vp1–2 and Vp3–4 PVTT, inconsistently use RECIST 1.1 or mRECIST, and apply nonstandardized criteria for surgical timing, perioperative treatment interruption, and postoperative adjuvant therapy. Therefore, prospective randomized trials comparing sequential surgery with continued nonsurgical therapy, together with standardized reporting of conversion-specific endpoints and prospective validation of biomarkers, are needed.^37^^,^^38^^,^^53^^,^^55^^,^^56^ Until such evidence is available, sequential surgery should remain an individualized multidisciplinary decision rather than a universal standard of care.

## 6 Decision-making and perioperative management for sequential surgery

### 6.1 Surgical window and perioperative management

From a surgical perspective, evaluation for conversion therapy should begin before treatment initiation rather than after tumor shrinkage. At baseline, the potential resection range, possibility of an inadequate future liver remnant, relationship between tumor thrombus and the portal vein, hepatic veins, or inferior vena cava, and whether extrahepatic lesions can be treated synchronously or sequentially should be defined by a multidisciplinary team. 15, 16, 17 Such preplanning is important because the surgical team can act immediately within the operative window once the expected treatment response is achieved. Treatment can be promptly adjusted to ablation, TACE consolidation, or continued systemic therapy if hepatic function declines or the distribution of residual disease changes during therapy. Conversely, without baseline surgical planning, patients may experience delays in preoperative preparation even after achieving radiographic resectability or miss the optimal window because of an excessive pursuit of a deeper response. Fig. 1 shows the proposed decision algorithm integrating the principles discussed in this review.

**Fig. 1:**
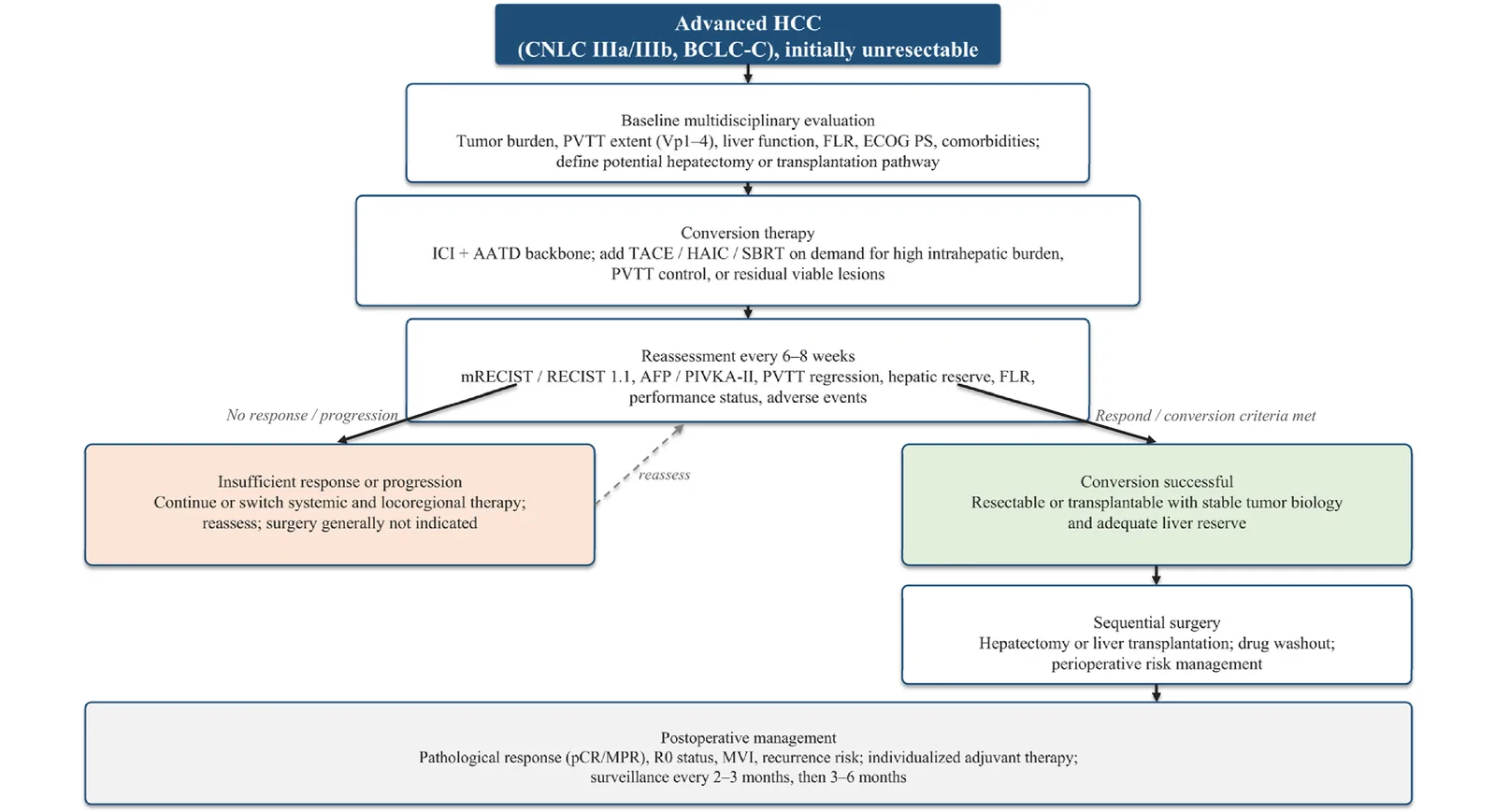
Proposed multidisciplinary algorithm for conversion therapy and sequential surgery in advanced HCC. Abbreviations: AATD, antiangiogenic targeted drug; AFP, alpha-fetoprotein; BCLC, Barcelona Clinic Liver Cancer; CNLC, China Liver Cancer Staging; ECOG PS, Eastern Cooperative Oncology Group performance status; FLR, future liver remnant; HAIC, hepatic arterial infusion chemotherapy; HCC, hepatocellular carcinoma; ICI, immune checkpoint inhibitor; MPR, major pathological response; mRECIST, modified Response Evaluation Criteria in Solid Tumors; MVI, microvascular invasion; pCR, pathological complete response; PIVKA-II, protein induced by vitamin K absence or antagonist-II; PVTT, portal vein tumor thrombosis; R0, complete macroscopic and microscopic resection; RECIST 1.1, Response Evaluation Criteria in Solid Tumors version 1.1; SBRT, stereotactic body radiotherapy; TACE, transarterial chemoembolization; Vp, portal vein invasion grade.

The surgical window should be defined as the period during which the tumor becomes resectable, tumor biology is controlled, liver function and general status can still tolerate surgery, and resistance has not yet emerged. Performing surgery too early may result in insufficient conversion, excessive resection, or residual viable tumor thrombus, whereas performing surgery too late may result in loss of opportunity due to acquired resistance, hepatic dysfunction, cumulative adverse events, or new metastases. In practice, multidisciplinary evaluation can be performed every 6–8 weeks after treatment initiation, integrating contrast-enhanced magnetic resonance imaging or computed tomography, RECIST 1.1 and mRECIST, AFP/PIVKA-II changes, hepatic reserve, nutritional status, and adverse events. 15, 16, 17^,^^35^

The determination of ideal surgical timing relies on the following conditions: target lesions exhibit complete or partial response or markedly reduced enhancement; portal vein or hepatic vein tumor thrombus regresses, becomes organized, or demonstrates improved relationship with the vessel wall; no new intrahepatic lesion or extrahepatic progression is present; AFP and PIVKA-II decrease substantially or reach a stable plateau; R0 resection is expected, and postoperative liver remnant volume, hepatic inflow and outflow, and biliary structures are preserved; Child–Pugh class A or carefully selected class B status is present, with acceptable ICG R15, platelet count, and coagulation function; and Eastern Cooperative Oncology Group performance status is 0–1 with American Society of Anesthesiologists class ≤ III. Timely surgery should be favored over pursuing a deeper radiographic response alone if two consecutive assessments indicate that efficacy has plateaued and the patient already meets resectability criteria. ^15^^,^^17^^,^^35^^,^^65^, ^66^, ^67^, ^68^, ^69^, ^70^

Perioperative drug management is important for sequential surgery safety. Bevacizumab and other anti-VEGF antibodies are associated with bleeding, thrombosis, and impaired wound healing and require discontinuation for at least 6 weeks before surgery. No unified standard exists for ICI discontinuation before surgery. Therefore, the decision should be individualized based on immune-related adverse events, hepatitis risk, and surgical urgency.^11^^,^^12^^,^^15^^,^^16^ For patients undergoing TACE, HAIC, or SBRT, surgery should be deferred until recovery from acute hepatic injury, biliary injury, myelosuppression, or radiation-related reactions. The overall principle is to complete surgical sequencing when the tumor is resectable, the liver function is optimal, and the drug-related risk is controllable.

### 6.2 Postoperative adjuvant therapy and long-term follow-up

Whether adjuvant therapy is needed after conversion therapy followed by sequential surgery should be determined according to the initial stage, treatment response, pathological findings, and tolerability rather than determined by a uniform rule. Patients with advanced HCC may remain at high risk of recurrence even after R0 resection after conversion because of initial PVTT, large tumor size, multifocal disease, history of extrahepatic metastasis, markedly elevated AFP/PIVKA-II, or postoperative residual viable tumor. Current evidence for traditional adjuvant therapy is inconsistent. The STORM trial failed to demonstrate the benefit of sorafenib after resection or ablation in a general postoperative population. The initial analysis of Imbrave050 showed improved RFS with atezolizumab plus bevacizumab in high-risk patients. However, the updated results showed that the early RFS benefit was not sustained (RFS HR, 0.90; 95% CI: 0.72–1.12), and the overall benefit-risk profile did not support routine adjuvant use. 71, 72, 73.

A tripartite decision framework that incorporates pathological response, recurrence risk, and treatment tolerability may be applied after conversion surgery. Close surveillance or short-course consolidation therapy may be considered if pCR or MPR is achieved, R0 resection is confirmed, microvascular invasion and satellite nodules are absent, tumor markers normalize, and preoperative treatment toxicities are prominent. In the presence of non-pCR, microvascular invasion, satellite nodules, poor differentiation, close or positive margin, residual tumor thrombus, or persistently abnormal tumor markers, continuation or adjustment of the effective preoperative combined ICIs and AATDs regimen after recovery may be considered, with TACE, radiotherapy, or other locoregional treatments added according to recurrence risk. Retrospective studies on very high-risk groups, such as those with PVTT, have shown that adjuvant targeted immunotherapy has a duration-benefit plateau. A multicenter retrospective study of patients who underwent R0 resection after successful conversion of initially unresectable HCC with PVTT showed that a 4–6-months course was noninferior to longer treatment and appeared to be the optimal window, but prospective confirmation is lacking.^74^ Whether a 6- to 12-month course adds benefit remains unproven. Therefore, treatment duration should be individualized according to pathological risk, tolerability, and patient preference, and fixed duration recommendations should await randomized evidence.^70^^,^^74^

Long-term postoperative management should include control of underlying liver disease and surveillance for recurrence. Patients with HBV-related HCC should receive standard antiviral therapy to control HBV DNA replication and hepatitis activity. Portal hypertension, malnutrition, ascites, and fluctuation in hepatic function should be managed in patients with cirrhosis. Imaging follow-up should be performed every 2–3 months during the first 2 postoperative years and then extended to every 3–6 months according to recurrence risk, whereas AFP, PIVKA-II, liver function, and toxicity of the ICI and AATD combination should be monitored simultaneously.^11^^,^^12^^,^^75^^,^^76^ The follow-up interval should be shortened, and contrast-enhanced magnetic resonance imaging, chest computed tomography, or positron emission tomography-computed tomography should be performed promptly if tumor markers rise before radiographic recurrence is detected, so that occult recurrence can be considered again for local radical therapy or systemic treatment.

## 7 Conclusions and perspectives

Systemic therapy for advanced HCC has entered an era in which the combination of ICIs and AATDs plays a central role. Conversion therapy and sequential surgery can help selected patients with initially unresectable disease regain the possibility of curative treatment. Existing evidence supports the combination of ICIs and AATDs as an important backbone for conversion therapy, with selective addition of TACE, HAIC, SBRT, and other locoregional treatments in appropriate clinical scenarios. Decisions regarding sequential surgery after successful conversion should be made based on oncologic benefit, hepatic functional reserve, future liver remnant volume, perioperative drug safety, and patient preference rather than on radiographic response alone. Postoperative adjuvant therapy remains unstandardized and should be tailored according to pathological response and risk of recurrence. This review synthesized recent clinical evidence, focused on conversion-specific endpoints, and provided a practical framework for translating treatment response into surgical decision-making. However, important questions remain regarding the selection of the optimal regimen, prediction of pathological response, definition of the surgical window, perioperative discontinuation strategy, duration of adjuvant therapy, and standards for recurrence monitoring. Given that most supporting surgical evidence is nonrandomized, the benefit of sequential surgery may partly reflect the selection of favorable tumor biology rather than a causal effect of surgery. Further multicenter prospective studies, real-world cohort studies, and translational research are needed to define patients most likely to benefit, standardize treatment pathways, and advance conversion therapy toward evidence-based, biomarker-guided precision care.

## CRediT authorship contribution statement

**Yuhao Huo:** Writing – original draft, Investigation, Formal analysis, Conceptualization. **Hongxiang Jiang:** Writing – original draft, Conceptualization, Investigation. **Yuanzhe Liu:** Software, Investigation. **Tong Jiang:** Investigation. **Cong Ren:** Investigation. **Xu Zhang:** Resources. **Boyu Wu:** Formal analysis. **Haowen Tang:** Writing – review & editing.

## Informed consent

Not applicable.

## Ethics statement

Not applicable. This review did not involve newly collected human or animal data.

## Organ donation

Not applicable.

## Animal treatment

Not applicable.

## Data availability statement

No new data were generated or analyzed in this review. All data discussed are available in the cited published literature.

## Declaration of generative AI and AI-assisted technologies in the manuscript preparation process

During the preparation of this work the author(s) used DeepSeek in order to improve the language and readability of the manuscript. After using this tool/service, the author(s) reviewed and edited the content as needed and take(s) full responsibility for the content of the published article.

## Funding

This research did not receive any specific grant from funding agencies in the public, commercial, or not-for-profit sectors.

## **Declarationofcompetinginterest**

The authors declare that they have no known competing financial interests or personal relationships that could have appeared to influence the work reported in this paper.
